# Assessing Adherence to Statin Prescription Guidelines at a Student-Run Free Clinic: A Retrospective Review

**DOI:** 10.7759/cureus.43083

**Published:** 2023-08-07

**Authors:** Alex Roetzheim, Sam Snow, Eduardo Gonzalez, Lucy Guerra, John Petrilli

**Affiliations:** 1 Department of Family Medicine, Florida State University College of Medicine, Tallahassee, USA; 2 Department of Family Medicine, University of South Florida Morsani College of Medicine, Tampa, USA; 3 Department of Internal Medicine, University of South Florida Morsani College of Medicine, Tampa, USA

**Keywords:** atherosclerotic cardiovascular disease, cardiovascular risk, primary prevention, hyperlipidemia, statin

## Abstract

Objective: The goals of this quality improvement project are to assess the BRIDGE Student-Run Free Clinic's adherence to the 2019 American College of Cardiology (ACC)/American Heart Association (AHA) Guideline on the Primary Prevention of Cardiovascular Disease and to compare our rate of statin prescription to the national average and to uninsured groups.

Methods: A quality improvement project of 205 patients qualified by initial inclusion criteria at a student-run free healthcare clinic. Socio-demographic information, clinical measures associated with cardiovascular risk, and documentation regarding statin prescription at the follow-up visit after a patient’s first lipid panel were abstracted from medical records. Descriptive statistics were calculated on the sample (proportions, means), and the proportion of patients eligible for statin treatment was determined.

Results: Of 58 patients eligible by guidelines to receive statins, 29 received a statin (50%) at their follow-up visit. Patients with clinical atherosclerotic cardiovascular disease (ASCVD) were more likely to receive statin therapy (83.3%) compared to other groups. Patients who were prescribed a statin were older, had higher total cholesterol, higher low-density lipoprotein (LDL), higher systolic blood pressure, and had higher ASCVD risk. Patients receiving statins were also more likely to be male or have a history of either hypertension, diabetes, or clinical ASCVD.

Conclusion: Patients with established ASCVD had high rates of statin prescription. Following the first lipid panel, clinicians prescribed statins to approximately 50% of eligible patients. Although this proportion is below the national average for insured patients, it is higher than the national average for uninsured patients and represents a relatively high proportion of eligible patients within the examined time frame.

## Introduction

The benefits of statins in cardiovascular therapy are well established in the medical literature [1]. The American College of Cardiology (ACC)/American Heart Association (AHA) cholesterol guidelines outline populations that have been shown to clearly benefit from statin prescription [2]. Criteria include (1) patients with clinical atherosclerotic cardiovascular disease (ASCVD), (2) patients with low-density lipoprotein (LDL) >190, (3) patients aged 40-75 with diabetes and LDL 70-189, and (4) patients aged 40-75 without clinical ASCVD or diabetes but with ASCVD risk >7.5%.

Despite significant increases in statin prescription in the last decade, there continue to be disparities in populations that receive appropriate statin therapy [3]. Previous research of a large healthcare system found that only 62.3% of patients eligible to receive statins were prescribed them [3]. Rates of statin prescription are lower in racial and ethnic minority populations [4]. Some studies report statin prescription rates in underserved and uninsured populations as low as 33.6% [4]. There is a significant gap in the literature pertaining to statin prescription rates in underserved populations compared to standard insured populations, especially among student-run free clinics (SRFCs). We hope to provide insight into current rates of statin prescriptions among underserved patients at an SRFC in the Southeastern United States. 

The goals of this quality improvement project are to assess our SRFC’s adherence to the 2019 ACC/AHA Guideline on the Primary Prevention of Cardiovascular Disease and to compare our rate of statin prescription to the national average and to uninsured groups. This quality improvement project strives to ensure that statins are prescribed appropriately, decrease missed treatment opportunities based on these guidelines, and improve the quality of care delivered to our patients. 

## Materials and methods

This quality improvement project utilized a retrospective medical record abstraction of Practice Fusion Electronic Health Records from the SRFC. Patients attending the clinic from the earliest available records in the electronic medical record to the most current date of chart review (July 2021) were eligible for inclusion. Clinic patients were eligible for inclusion if they were aged 40-75, had at least two clinic visits, and were potentially eligible for statin therapy (documented LDL cholesterol 70 mg/dL or greater). We determined statin guideline adherence among patients if evidence of a statin prescription was found in the medication list or clinical progress notes. All data for this project were collected from the first follow-up visit after a patient’s first documented lipid panel.

A total of 461 patients were assessed for eligibility and 256 patients were excluded from the sample (clinical data missing needed for ASCVD calculation (n=47), less than two patient encounters (n=152), patients not in the eligible age range (n=38), LDL cholesterol less than 70 mg/dL (n=10), duplicate or missing medical record (n=9)), leaving 205 eligible patients. This represents the entire clinic patient population meeting eligibility criteria, not a subsample of patients.

Two trained medical students abstracted medical records on the remaining 205 eligible patients. Socio-demographic information was collected (age, gender, race-ethnicity) along with clinical measures associated with cardiovascular risk (blood pressure, lipid values, and prior history of cardiovascular disease/diabetes/hypertension) and documentation regarding statin prescription. Of the 205 eligible patients, at the time of their first documented lipid panel reading, 58 met the ACC/AHA guideline criteria for statin use.

Using Statistical Analysis Software (SAS 9.4, Cary, North Carolina), descriptive statistics were calculated on the sample (proportions, means), and the proportion of patients eligible for statins and prescribed statins was determined. Factors associated with statin use were assessed with the chi-square test for categorical variables and with Student’s t-test for continuous measures. For continuous variables, we confirmed normality visually by inspecting frequency distributions and measures of skewness and kurtosis. All p-values are two-tailed, and alpha was set at 0.05.

## Results

Patient clinical characteristics are reported in Table 1. The mean age of the sample was 55.8 years (SD 7.3 years), and the sample was primarily female (70.7%). Among patients for whom ethnicity was recorded, the sample was overwhelmingly Hispanic (91.6%). Sizeable percentages of the sample had either pre-existing ASCVD (8.8%), hypertension (31.2%), or diabetes (14.2%). 

**Table 1:** Selected cardiovascular-related clinical characteristics of the study population (n=205). HDL: high-density lipoprotein; LDL: low-density lipoprotein; BP: blood pressure; ASCVD: atherosclerotic cardiovascular disease.

Characteristics	Mean	SD
Age (years)	55.8	7.3
Total cholesterol (mg/dL)	199.4	39.5
HDL (mg/dL)	51.0	13.6
LDL (mg/dL)	121.8	31.0
Systolic BP (mmHg)	127.9	16.6
ASCVD (10-year risk %)	4.5	5.9

Among the sample of 205 patients, clinicians prescribed statins to 39 patients (19.0%), including 10 patients for whom the guideline criteria were not met. Factors associated with statin use among the 205 patients included gender (male 28.3% vs. female 15.2%, p=0.03), ethnicity (non-Hispanic 38.5% vs. Hispanic 15.5%, p=0.04), treatment of hypertension (39.1% vs. 9.9%, p<0.001), diabetes (44.8% vs. 14.8%, p<0.001), or prior ASCVD (83.3% vs. 12.8%, p<0.001). Patients who were prescribed statins were older (mean age 60.5 years vs. 54.7 years, p<0.001), had higher values of total cholesterol (mean 228.0 mg/dL vs. 192.7 mg/dL, p<0.001), higher values of LDL cholesterol (mean 145.5 mg/dL vs. 116.3 mg/dL, p<0.001), higher systolic blood pressures (mean 135.4 mmHg vs. 126.1 mmHg, p=0.001), and had higher 10-year predicted ASCVD risk (10.0% vs. 3.3%, p<0.001). Statin prescriptions based on patient characteristics are reported in Table 2.

**Table 2:** Statin prescriptions in relation to patient characteristics. ASCVD: atherosclerotic cardiovascular disease. Numbers may not total 205 due to missing data.

Variable	All patients (n=205)	Statin prescribed	p-value
Sex			0.03
Male	60	17/60 (28.3%)	
Female	145	22/145 (15.2%)	
Race			0.63
African American	18	4/18 (22.2%)	
White	82	12/82 (14.6%)	
Other	23	5/23 (21.7%)	
Undecided	82	18/82 (22.0%)	
Smoker			0.30
Yes	21	5/21 (23.8%)	
No	161	27/161 (16.8%)	
Former	20	6/20 (30%)	
Treatment hypertension			<0.001
Yes	64	25/64 (39.1%)	
No	141	14/141 (9.9%)	
History of ASCVD			<0.0001
Yes	18	15/18 (83.3%)	
No	187	24/187 (12.8%)	
Diabetes			<0.0001
Yes	29	13/29 (44.8%)	
No	176	26/176 (14.8%)	

Overall, 58 (28.3%) of the 205 patients met the guideline criteria for statin prescription, and among this group, 29 (50.0%) were taking or prescribed a statin at the first visit after their first lipid panel. Patients with established ASCVD were more likely to be prescribed statins (83.3%) than patients with other guideline indications (diabetes: 37.5%; LDL > 190 mg/dL: 0%; 10-year ASCVD risk >7.5%: 33.3%). Statin prescriptions based on 2019 ACC/AHA guidelines are reported in Table 3.

**Table 3:** Statin prescriptions based on 2019 ACC/AHA guideline criteria. ASCVD: atherosclerotic cardiovascular disease; LDL: low-density lipoprotein. p-values are for comparisons between the guideline indication group and all other patients.

Variable	Eligible	Statin prescribed	p-value
History of ASCVD	18	15 (83.3%)	<0.0001
LDL >190	1	0 (0%)	0.63
Diabetes	24	9 (37.5%)	0.01
ASCVD >7.5%	15	5 (33.33%)	0.14
Total Eligible	58	29 (50.0%)	<0.0001

## Discussion

Our project provides insight into statin prescription rates at an SRFC. We found that our clinic attained a “first pass” statin prescription rate of 50% among those meeting 2019 ACC/AHA guidelines. This rate falls short of the national average of 62.3% in an insured population [3], but it is well above the 33.6% documented in uninsured patients and was based solely on the first visit following a patient’s first documented lipid panel [4]. At the first opportunity to prescribe statins, more than half of the indicated patients received statins. Larger studies typically report statin prescription rates over a period of months, which may account for higher prescription rates when compared to our data. 

Our clinic excelled in prescribing statins to patients with a history of ASCVD. We found that patients with ASCVD received statins at higher rates than all other guideline criteria groups. We prescribed at a rate of 83.3%, which far surpassed the national averages for underserved populations with ASCVD (38%) [5] and was comparable to the general, both insured and uninsured, national average statin prescription rate for those with ASCVD (83.9%) [3]. Overall, patients who were prescribed statins also tended to have diabetes and higher LDL cholesterol. Lastly, the analysis showed a tendency to prescribe statins more to men versus women, which is consistent with current literature [3].

Multiple reasons may exist for why statins are not more readily prescribed when clinically indicated. As seen in other free clinics, the unique socioeconomic circumstances relating to poverty, low health literacy, immigration, and language barriers can have a detrimental impact on medication initiation and adherence [6]. As a result, mutual decision-making between the patient and physician may lead to prioritizing only one or two crucial medications to optimize adherence. Statin prescriptions may therefore be delayed when acute needs are prioritized, particularly for patients suffering from diabetes or who qualify solely based on their ASCVD risk. 

Another reason for delayed prescribing is “clinical inertia,” which is the phenomenon of delaying or failing to initiate therapy when indicated [7]. This phenomenon is a well-recognized barrier to improving patient care and clinical outcomes [7]. Continued focus on lifestyle modifications may explain the lower prescription rates among patients who are recommended to start a statin based solely on their ASCVD risk calculation.

Although the ACC/AHA criteria provide the framework for the treatment and prevention of cardiovascular disease, the guidelines have been criticized [8] and some clinicians may not agree with them. Clinicians concerned about overprescribing statins may engage healthy patients in mutual decision-making, ultimately deciding not to pursue statin therapy [9].

The next steps to improving the quality of care include ensuring that cardiovascular risk information needed by clinicians is recorded in a timely and visible manner within the electronic health records. Additionally, patient education is crucial to ensuring their understanding of the benefits of CV risk reduction, helping patients navigate medical misinformation found online, and specifically tackling cultural and language barriers in this population [10,11]. As guidelines evolve, making these criteria clear to clinic staff should also improve prescription rates. Given the interdisciplinary nature of our SRFC, involving pharmacy students in team-based care may decrease the clinical inertia of statin prescriptions, as shown in previous studies [7]. Our goals moving forward strive to eliminate barriers to statin prescription and provide better quality of care to our patients. 

Limitations for our project include our assessment of statin prescriptions at the first follow-up visit. Some patients may have received statin prescriptions at subsequent visits. In addition, statin prescriptions were assessed strictly within the medical records, and prescriptions not recorded in the medical record or occurring in other systems of care would be missed. Finally, patients may also have been on a statin when the first lipid panel was obtained, which would impact ASCVD calculations and may explain why some patients were prescribed statins despite not meeting ASCVD guidelines.

## Conclusions

In summary, we continue to see disparities in statin prescription among underserved and minority populations, including those cared for at SRFCs. Patients most in need of statins (those with established ASCVD) had high rates of prescription, and the overall rate of statin prescriptions for those with indications was 50%. Future studies should be done to examine statin prescriptions longitudinally and to assess patients’ actual compliance with statins in order to better understand the challenges of meeting ACC/AHA guidelines for care at SRFCs.
